# Compilation and functional classification of telomere length-associated genes in humans and other animal species

**DOI:** 10.18699/VJGB-23-34

**Published:** 2023-06

**Authors:** E.V. Ignatieva, N.S. Yudin, D.M. Larkin

**Affiliations:** Institute of Cytology and Genetics of the Siberian Branch of the Russian Academy of Sciences, Novosibirsk, Russia; Institute of Cytology and Genetics of the Siberian Branch of the Russian Academy of Sciences, Novosibirsk, Russia; Institute of Cytology and Genetics of the Siberian Branch of the Russian Academy of Sciences, Novosibirsk, Russia

**Keywords:** telomere length, candidate genes, genome-wide association study, functional classification, длина теломер, гены-кандидаты, полногеномный анализ ассоциаций, функциональный анализ

## Abstract

Telomeres are the terminal regions of chromosomes that ensure their stability while cell division. Telomere shortening initiates cellular senescence, which can lead to degeneration and atrophy of tissues, so the process is associated with a reduction in life expectancy and predisposition to a number of diseases. An accelerated rate of telomere attrition can serve as a predictor of life expectancy and health status of an individual. Telomere length is a complex phenotypic trait that is determined by many factors, including the genetic ones. Numerous studies (including genome-wide association studies, GWAS) indicate the polygenic nature of telomere length control. The objective of the present study was to characterize the genetic basis of the telomere length regulation using the GWAS data obtained during the studies of various human and other animal populations. To do so, a compilation of the genes associated with telomere length in GWAS experiments was collected, which included information on 270 human genes, as well as 23, 22, and 9 genes identified in the cattle, sparrow, and nematode, respectively. Among them were two orthologous genes encoding a shelterin protein (POT1 in humans and pot-2 in C. elegans). Functional analysis has shown that telomere length can be influenced by genetic variants in the genes encoding: (1) structural components of telomerase; (2) the protein components of telomeric regions (shelterin and CST complexes); (3) the proteins involved in telomerase biogenesis and regulating its activity; (4) the proteins that regulate the functional activity of the shelterin components; (5) the proteins involved in telomere replication and/or capping; (6) the proteins involved in the alternative telomere lengthening; (7) the proteins that respond to DNA damage and are responsible for DNA repair; (8) RNA-exosome components. The human genes identified by several research groups in populations of different ethnic origins are the genes encoding telomerase components such as TERC and TERT as well as STN1 encoding the CST complex component. Apparently, the polymorphic loci affecting the functions of these genes may be the most reliable susceptibility markers for telomere-related diseases. The systematized data about the genes and their functions can serve as a basis for the development of prognostic criteria for telomere length-associated diseases in humans. Information about the genes and processes that control telomere length can be used for marker-assisted and genomic selection in the farm animals, aimed at increasing the duration of their productive lifetime

## Abstract (ru)

Теломеры – это концевые участки хромосом, обеспечивающие их стабильность в ходе клеточного деления. Укорочение теломер инициирует процесс старения клеток, что может приводить к дегенерации и атрофии тканей. Укорочение теломер связано с сокращением продолжительности жизни и с предрасположенностью к ряду заболеваний, поэтому данный показатель может быть использован в качестве предиктора продолжительности жизни и состояния здоровья отдельного индивида. Длина теломер – сложный фенотипический признак, который определяется многими факторами, в том числе генетическими. Многочисленные исследования (включая полногеномный анализ ассоциаций, ПГАА) свидетельствуют о полигенном характере контроля длины теломер. Цель работы – охарактеризовать генетические основы регуляции длины теломер на основе данных ПГАА, полученных при исследовании различных популяционных выборок человека и других животных. Для этого авторами была собрана компиляция генов, ассоциированных с длиной теломер по данным ПГАА, которая включала сведения о 270 генах человека, а также 23, 22 и 9 генах, выявленных у крупного рогатого скота, домового воробья и нематоды соответственно. Среди них присутствовали два гена-ортолога, кодирующих белок шелтеринового комплекса (POT1 у человека и pot-2 у C. elegans). Функциональный анализ показал, что на длину теломер могут влиять генетические варианты в генах, кодирующих: 1) структурные компоненты теломеразы; 2) белковые компоненты теломерных участков хромосом (шелтериновый комплекс и CST комплекс); 3) белки, участвующие в биогенезе теломеразы и регулирующие ее активность; 4) белки, регулирующие функциональную активность компонентов шелтеринового комплекса; 5) белки, участвующие в репликации и/или кэпировании теломер; 6) белки, контролирующие альтернативный путь удлинения теломер; 7) белки, реагирующие на повреждения ДНК и отвечающие за репарацию; 8) компоненты РНК экзосом. В работе выявлены гены человека, идентифицированные несколькими исследовательскими группами в популяциях различного этнического происхождения. Это гены, кодирующие компоненты теломеразы (TERC и TERT), а также ген STN1, кодирующий белок CST комплекса. По-видимому, полиморфные локусы, затрагивающие функции этих генов, могут быть наиболее надежными маркерами предрасположенности к заболеваниям, связанным с длиной теломер. Систематизированные нами данные о генах и их функциях будут полезны при разработке прогностических критериев заболеваний человека, для которых показана связь с длиной теломер. Сведения о генах и процессах, контролирующих длину теломер, могут быть востребованы для маркер-ориентированной и геномной селекции сельскохозяйственных животных, направленной на повышение продолжительности их хозяйственного использования.

## Introduction

**Fig. 1.**
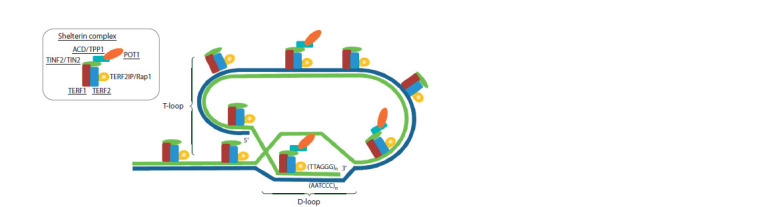
Structure of a chromosome’s telomeric region Telomeric DNA is presented as a T-loop reconstructed following the black-and-white illustration from Fan and co-workers (2021); nucleotide sequences in the DNA strands are not shown (Fan et al., 2021). The top left is simplified illustration of the relative positions of shelterin subunits, following the description from Jafri and co-workers (2016). Since the ACD/TPP1 and POT1 are much less abundant in nuclei (de Lange, 2018), some shelterin complexes are depicted without these subunits. D-loop is a structure, where two strands of the double-stranded DNA are separated, and one of them connects with the third DNA strand (a single-stranded 3’-end of the telomeric DNA region). The names of proteins corresponding to the human genes associated with telomere length according to the GWAS data are underlined

Telomeres are the terminal regions of chromosomes that ensure their stability and represented by evolutionary conserved tandemly repeated DNA sequences (e. g., a hexanucleotide TTAGGG repeat in vertebrates) of several kb in length (Podlevsky, 2008; Monaghan, Ozanne, 2018). For example, their lengths in humans at birth are 10–15 kb (Jafri et al., 2016). 3ʹ terminal end of a telomere is a single-stranded guanine-rich DNA region (150–200 nucleotides), whose end interacts with the double-stranded region to form the so-called T-loop at the telomere end. T-loop formation and stabilization are ensured by a shelterin complex (Fig. 1). This structure prevents recognition of a chromosome terminal region by repair proteins (de Lange, 2018).

DNA polymerase is unable to fully replicate the 3ʹ-end of a linear DNA during cell division, which leads to a loss of 50–200 nucleotides of the telomeric sequence at each cell division (Fan et al., 2021). Telomere shortening can also be facilitated by other factors and processes (Suppl. Material 1)1, such as oxidative stress, inflammation, UV irradiation, effects of toxic agents, DNA replication errors, etc. (Aviv, Shay, 2018; Monaghan, Ozanne, 2018). These factors are likely to produce different effects depending on cell types and the organism’s development stage and species (Monaghan, Ozanne, 2018).

Supplementary Materials are available in the online version of the paper: http://vavilov.elpub.ru/jour/manager/files/Suppl_Ignatieva_Engl_27_3.pdf.


Telomere shortening initiates cellular senescence. Activation of DNA damage response signaling pathways results in a cell cycle arrest, which may in turn lead to apoptosis and eventually – to progressive tissue degeneration (Jafri et al., 2016; Aviv, Shay, 2018; Monaghan, Ozanne, 2018). The data collected at the cellular level in vitro and in model organisms lay the foundation for using the telomere length as a predictor of life expectancy and health status of an individual. Indeed, studies in humans (Crocco et al., 2021), mice (Vera et al., 2012), sheep (Wilbourn et al., 2018), cattle (Seeker et al., 1 Supplementary Materials 1–11 are available in the online version of the paper: http://vavilov.elpub.ru/jour/manager/files/Suppl_Ignatieva_Engl_27_3.pdf. 2021), wild birds (Bichet et al., 2020), and other animals have shown that shorter telomere length may be associated with reduced life expectancy. The studies in humans discovered an association between the telomere length and cardiovascular diseases, cancer, diabetes, inflammation, and other pathological states (Kong et al., 2013; Jafri et al., 2016; Aviv, Shay, 2018).

Telomere shortening is prevented by telomerase, a specialized ribonucleoprotein complex acting as a reverse transcriptase. In humans, telomerase is active in almost all the cancer cells studied (Jafri et al., 2016), in blastocyst, in most somatic tissues at 16–20 weeks of development (except for brain cells), and ovary and sperm cells at all ontogenetic stages (except for mature spermatozoids and oocytes) (Wright et al., 1996).

**Fig. 2.**
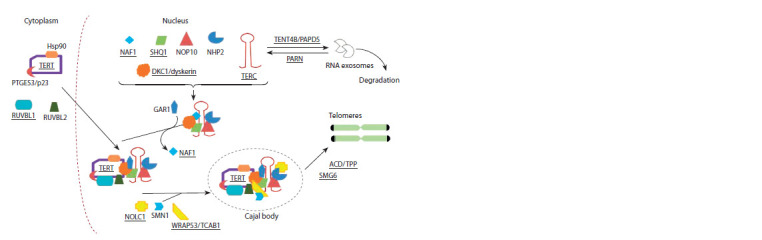
Simplified presentation of the main stages of telomerase biogenesis. The names of proteins and RNAs corresponding to the human genes associated with telomere length (as per GWAS results) are underlined. The scheme is based on the data on protein functions from the research articles cited in Suppl. Material 2

**Fig. 3.**
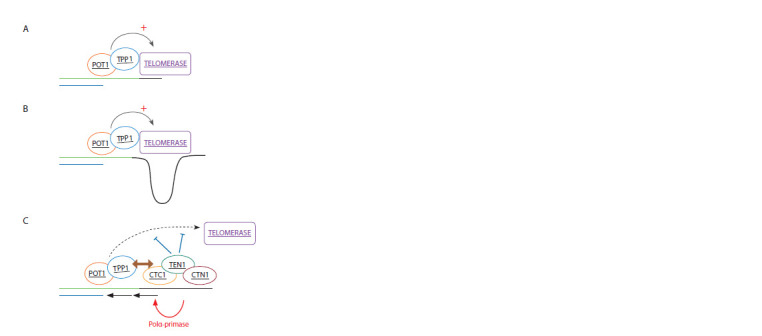
The role of CST proteins in telomerase activity regulation at the late S/G2 phase The first step of the five-step mechanism described by Chen et al. (2012) is the recruitment of telomerase and additional ACD/TPP1 and POT1 by shelterin complex (Step 1, Recruitment, not shown in the figure). Then, telomerase starts extending the single-stranded region of the DNA molecule (Step 2, Extention I ) (shown in Panel A, the newly synthesized DNA region is represented by a black line). After that (Step 3, Extention II ) the single-stranded region of the DNA molecule is further extended (see Panel B). CST proteins interact with the newly synthesized single-stranded DNA region (~60 nucleotides) hindering the stimulating effect of ACD/TPP1 and POT1 on telomerase (Step 4, Termination) and initiating C-strand synthesis by DNA polymerase alpha-primase (Polα- primase) (Step 5, Fill-in). Steps 4 and 5 are presented in Panel C. The names of proteins corresponding to the human genes associated with telomere length (as per GWAS results) are underlined.

Telomerase activity is controlled by the proteins regulating expression of telomerase components, their movement to various cell compartments, processing, and assembly as well as by the proteins maintaining stability of the telomerase complex or, on the contrary, activating its degradation (Egan, Collins, 2012; Tseng et al., 2015; Schrumpfová, Fajkus, 2020). The main stages of telomerase biogenesis are presented in Figure 2. The examples of proteins affecting the telomerase activity are presented in Suppl. Material 2. In addition, the telomerase activity is also affected by the shelterin (Diotti, Loayza, 2011; de Lange, 2018) and CST complex (Fig. 3) (Chen et al., 2012).

Telomere length is a complex phenotypic trait determined by multiple factors including genetic ones. The meta-analysis of heritability data for this trait performed in eighteen vertebrate species showed the averaged heritability index of 45 %. The studies showed the value of 52 % in humans, 42 % – in Holstein cattle breed, 35 % – in hamadryas baboons, and 5 % – in sheep (Chik et al., 2022).

The problem of genetic basis of telomere length regulation is of interest for many researchers. The information on telomerase components and proteins involved in telomere length regulation (including 20 proteins identified in mammals) can be found in The Telomerase Database (http://telomerase.asu. edu/) (Podlevsky et al., 2008). Joyce and co-workers (2018) presented a set of 80 human genes with telomere-related functions (Joyce et al., 2018).

The GWAS data also indicate a polygenic nature of telomere length heritability. For instance, the GWAS Catalog (https:// www.ebi.ac.uk/gwas/) cites 99 human genes that either include or neighbor the telomere length-associated allelic variants. One of the largest GWA studies presents the data on 138 human genomic loci, whose allelic variants are associated with telomere length (Codd et al., 2021).

In addition to the telomere length association data gathered using GWA studies in various human population samples, the data obtained in other animal species also appear to be of interest. However, these studies are rather scarce and are available only for Holstein–Friesian cattle (Ilska-Warner et al., 2019), house sparrow nestlings (Pepke et al., 2021), and C. elegans (Cook et al., 2016).

The objective of this review was to characterize the genetic basis of telomere length regulation using the GWAS data collected in various human populations and to compare them with the results of similar experiments in other animal species. For this purpose, (1) the data on genes identified in GWAS telomere length experiments were systematized; (2) functional annotation of genes was performed, and the set of biological processes affecting telomere length was identified.

## Materials and methods

The data on telomere length-associated genes were obtained from the papers available in the PubMed database (https:// pubmed.ncbi.nlm.nih.gov/) using such keywords as ‘telomere length’ and ‘GWAS’. Functional annotation of genes was performed using information obtained from the papers presenting GWAS data, PubMed, The Telomerase Database (http:// telomerase.asu.edu/) queries, and the DAVID knowledgebase (https://david.ncifcrf.gov/) (Sherman et al., 2022).

## Results and discussion

Human genes identified through GWA studies

PubMed queries produced 18 scientific papers presenting the results of identifying telomere length-associated polymorphic loci in human genome based on GWAS data. These papers were analyzed, and the data on 270 telomere length-associated genes were collected (Suppl. Material 3). Most genes (262) were identified in European-ancestry population samples, the data on 15 genes were obtained from the studies in Southeast Asian population samples (natives of China, Bangladesh, and India), five genes were identified as a result of trans-ethnic meta-analysis of Singaporean Chinese and European ancestry data (Dorajoo et al., 2019), and one gene was found in African Americans (Zeiger et al., 2018).

The data on functional significance in the context of telomere length regulation were presented by the authors of GWA studies for 52 genes out of 270 (see Suppl. Material 3). The fact that the data on gene significance in the context of telomere length regulation were unavailable for a number of loci reflects the capabilities and limitations of GWAS methodology. Most loci identified by GWAS and associated with the trait of interest are located in intergenic regions. As a rule, in these cases, the set of candidate genes includes the nearest genes, whose functional significance is often difficult to interpret. To identify the mechanisms and genes, through which intergenic variants affect the studied traits, additional experiments are required. For example, it was shown that T-to-C substitution of rs1421085 in the intron of FTO gene affects the expression of IRX3 and IRX5, whose transcription start sites are far away (~520 and ~1160 kb) from rs1421085 (Claussnitzer et al., 2015).

Main functional groups of human telomere length-associated genes

**Fig. 4.**
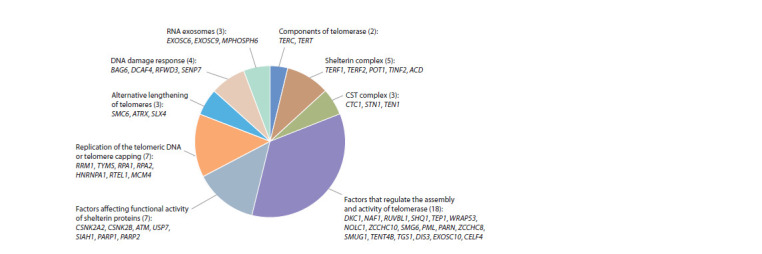
Functional groups of human telomere length-associated genes The classification is presented for 52 genes, whose role in telomere length regulation is characterized in Suppl. Material 4. The numbers given in parentheses indicate the numbers of genes in groups.

A functional classification was performed for a set of 52 human genes for which there was information about their functional significance in the context of telomere length regulation (Suppl. Material 4). As a result, several functional groups of genes have been identified (Fig. 4)

Genes encoding telomerase components: TERC is the telomerase RNA component acting as a matrix for DNA strand extension at the telomere end and TERT is a reverse transcriptase enzyme subunit (Egan, Collins, 2012; Tseng et al., 2015).

Genes encoding shelterin proteins: components of this complex (TERF1/TRF1, TERF2/TRF2, POT1, TERF2IP/ Rap1/DRIP5, TINF2/TIN2, and ACD/TPP1/TINT1) can bind to both double-stranded and single-stranded telomeric DNA regions (see Fig. 1), stabilize them, protect them from exonucleases, reduce telomerase access, and inhibit the proteins activated by damaged DNA and involved in double-stranded break repair (Diotti, Loayza, 2011; de Lange, 2018). The GWAS data on telomere length association were obtained for the genes coding for five out of six shelterin proteins (TERF1, TERF2, POT1, TINF2 and ACD/TPP1/TINT1) (see Fig. 4, Suppl. Material 4).

Genes encoding CST proteins: CTC1, STN1, TEN1. CST complex acts as a telomerase negative regulator at the late S to G2 phase of the cell cycle (see Fig. 3) (Chen et al., 2012).

Genes encoding proteins involved in telomerase biogenesis and regulating its activity. One of these genes, ZCCHC10, encodes a protein regulating telomerase synthesis at transcriptional level: ZCCHC10 suppresses TERT transcription (Ohira et al., 2019). Processing and assembly of a telomerase RNA subunit involves DKC1, NAF1, and SHQ1 (Egan, Collins, 2012), ribonuclease PARN, exoribonuclease DIS3, the component of a nuclear exosome targeting (NEXT) complex, ZCCHC8 (Tseng et al., 2015), SMUG1 (Kroustallaki et al., 2019), and CELF4/BRUNOL4 (Mangino et al., 2009). Noncanonical polymerase TENT4B/PAPD5 (Nagpal et al., 2020), trimethylguanosine synthetase TGS1 (Chen et al., 2020), and EXOSC10 RNA exosome component (Stuparević et al., 2021) cause a decrease in the level of active TERC. The assembly of telomerase nucleoprotein complex involves ATPase RUVBL1/ pontin (Jafri et al., 2016) and telomerase-associated protein TEP1 (Codd et al., 2021). Two proteins (WRAP53/ WDR79/TCAB1 and NOLC1/NOPP140) provide telomerase accumulation in Cajal bodies, the small nuclear organelles where processing of small nuclear and nucleolar RNAs and assembly or ribonucleoprotein complexes occur (Bizarro et al., 2019; Schrumpfová, Fajkus, 2020). Telomerase activity is modulated by activator protein SMG6/EST1A, which also binds to a single-stranded DNA (Snow et al., 2003), and PML protein, whose isoform PML-IV suppresses telomerase activity (Oh et al., 2009).

Genes encoding proteins regulating functional activity of shelterin proteins. CSNK2A2 and CSNK2B are the subunits of casein kinase which phosphorylates TERF1, increasing its binding to telomeres (Saxena et al., 2014; Li et al., 2020). ATM serine/threonine kinase, on the contrary, decreases TERF1 binding to the telomeric DNA (Li et al., 2020). Peptidase USP7 and ubiquitin ligase SIAH1 activate proteasomal degradation of POT1 and TERF2, respectively (Codd et al., 2021). ADP ribosylases PARP1 and PARP2 reduce the DNA binding activity of TERF2 (Dorajoo et al., 2019; Codd et al., 2021).

Genes encoding proteins involved in telomere replication and/or capping: (1) enzymes RRM1 and TYMS involved in synthesis of deoxynucleoside triphosphates (dNTP) and thymidylates required for DNA synthesis (Dorajoo et al., 2019; Nersisyan et al., 2019); (2) helicases RTEL1 and MCM4 (Codd et al., 2013, 2021); (3) RPA1 and RPA2, the subunits of the RPA complex capable to unfold G-quadruplex structures that may block DNA replication (Codd et al., 2021); (4) HNRNPA1 promoting telomere capping after DNA replication (Codd et al., 2021).

Genes encoding proteins affecting the alternative telomere lengthening pathway. This telomerase-independent mechanism (ALT or Alternative Lengthening of Telomere, see the description in Suppl. Material 5) includes the recombination between telomeric regions of two DNA molecules (Sobinoff, Pickett, 2017, 2020). Three genes identified in GWA studies were attributed to this group (see Fig. 4, Suppl Material 4). These genes encode SMC6 which activates ALT (Potts, Yu, 2007) and its two inhibiting proteins: ATRX with chromatin remodeling activity and SLX4 endonuclease (Sobinoff, Pickett, 2017).

Genes encoding DNA damage response proteins: (1) peptidase SENP7 (Li et al., 2020); (2) chaperone protein BAG6 (Li et al., 2020); (3) DCAF4 interacting with CUL4-DDB1 ligase (Mangino et al., 2015); (4) RFWD3 interacting with RPA protein (replication protein A) (Li et al., 2020).

Genes encoding subunits of RNA exosomes: EXOSC6, EXOSC9 (Codd et al., 2021) and MPHOSPH6 (Dorajoo et al., 2019). These proteins are functionally significant, because it is known that TERC may be subjected to 3ʹ-processing, and the RNA-exosomes are involved in this process (Tseng et al., 2015).

**Fig. 5.**
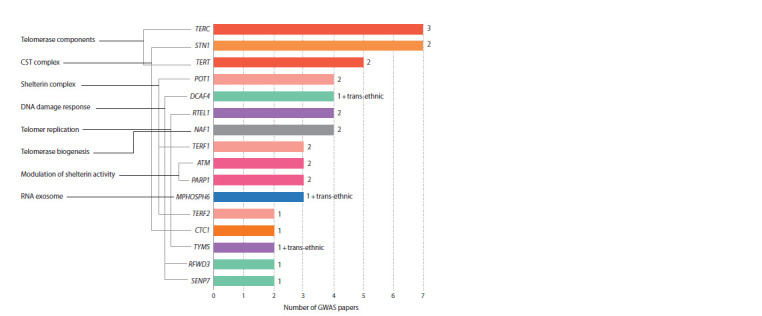
Genes revealed in at least two GWAS papers Colors of columns indicate functional groups the genes belong to. The numbers on the right of columns represent the number of ethnically diverse GWAS population samples, in which the genes were identified. Trans-ethnic is the group consisting of Singaporean Chinese and Europeans.

Human candidate genes identified in more than one study As mentioned above, we have analyzed 18 papers on identifying telomere length-associated human genome loci based on GWA studies and collected the data on 270 such genes (see Suppl. Material 3). Notably, only 16 genes were identified in at least two studies (Fig. 5).

The most frequently identified genes were the ones encoding both telomerase components (TERC and TERT ) and STN1 encoding a component of the CST complex (revealed in 7, 5, and 7 studies, respectively). Three genes POT1, TERF1, and TERF2 encoding components of the shelterin complex were mentioned in 4, 3, and 2 publications, respectively. Three more genes DCAF4, RTEL1, and NAF1 controlling the DNA damage response, telomere replication, and telomerase biogenesis were identified in four studies. ATM, PARP1, MPHOSPH6, RFWD3, SENP7, and TYMS were identified in 3 or 2 papers.

Most of 16 genes listed above were identified in population samples of different ethnic origin: (1) TERC in three ethnic groups, namely Europeans, Bangladeshis, and Singaporean Chinese; (2) DCAF4, MPHOSPH6, and TYMS in Europeans and as a result of the trans-ethnic meta-analyses (Singaporean Chinese+Europeans); (3) TERT, STN1, POT1, RTEL1, NAF1, TERF1, ATM, PARP1 in two ethnic groups, namely Europeans and Singaporean Chinese.

Identification of the genes related to telomere length regulation according to DAVID

Using DAVID, we found the terms from the GOTERM_BP_ DIRECT dictionary that were significantly (FDR < 0.05) associated with the list of 270 human genes presented in Suppl. Material 3. Sixteen terms indicating biological processes that directly control telomere length are presented in Suppl. Material 6, and the remaining fifteen terms are listed in Suppl. Material 7. There were 30 genes associated with the terms from the first group (see Suppl. Material 6), with two of them (SIRT6 and TP53) previously not recognized as biologically interpretable (these genes were presented in (Codd et al., 2021) without comment on their functional significance in the context of telomere length regulation). The analysis of scientific papers showed that proteins encoded by both genes can function in the subtelomeric regions of chromosomes (Tennen et al., 2021; Tutton et al., 2016), which means they could be indirectly involved in telomere length regulation

Then, the genes associated with the second group of GOTERM_ BP_DIRECT terms identified at FDR< 0.05 (see Suppl. Material 7) were analyzed. Among them, 29 genes were found that had no biological interpretation (highlighted in red in Suppl. Material 7, and listed in Suppl. Material 8). This group of 29 genes included the above mentioned SIRT6 and TP53, as well as BRCA1, SAMHD1, and BRCC3 associated with the maximum number of GO terms (six, four, and four, respectively). Apparently, the genes from the list thus obtained may also be of interest in the context of telomere length regulation.

Telomere length-associated genes found in other animal species

Genome-wide search for telomere length-associated loci and genes was carried out in three animal species: cattle (Bos taurus), sparrows (Passer domesticus), and nematodes (Caenorhabditis elegans).

A GWA study to investigate the species Bos taurus was carried out on 702 animals of the Holstein–Friesian breed (Ilska-Warner et al., 2019). The study of the DNA isolated from the whole blood of cows sampled at birth showed six telomere length-associated polymorphic loci, and three additional loci were identified when analyzing the DNA from blood samples at the first lactation. An analysis of the quantitative trait loci (QTL) corresponding to the identified genetic variants revealed 14 candidate genes at birth and 9 at the first lactation (see the Table and Suppl. Material 9). The authors were unable to find any data on direct involvement of the identified genes in processes associated with telomere length regulation. NUP93 nucleoporin gene encoding a nuclear pore component was considered a potential regulator, because it was shown earlier in yeast that nucleoporins facilitated silencing of genes in proximity of telomeric regions (Van de Vosse et al., 2013).

**Table 1.**
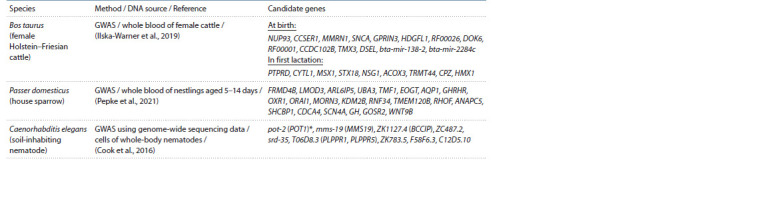
Telomere length-associated animal genes (see Suppl. Materials 9–11 for additional data) * C. elegans genes are cited with human orthologs in parentheses.

The recently published results of GWA study (Pepke et al., 2021) in house sparrow (Passer domesticus) nestlings made it possible to identify 22 candidate genes (see the Table and Suppl. Material 10). According to the authors, the genes of interest in the context of telomere length regulation seem to be as follows: (1) WNT9B encoding a protein component of Wnt/ β-catenin signaling pathway due to β-catenin involvement in Tert activation in embryonic stem cells of mice; (2) CDCA4, GH, and GHRHR regulating cell proliferation, apoptosis, and body growth; (3) RHOF involved in cytoskeletal organization; (4) RNF34 (E3 ubiquitin-protein ligase RNF34) regulating ubiquitination; (5) AQP1 due to involvement of aquaporin protein in transport of nitrogen oxide and active forms of oxygen, which increases oxidative stress which can in turn affect telomerase activity; (6) SCN4A, because its expression in human stem cells correlates with telomere length.

Our analysis showed that none of the candidate genes identified in cattle (23 genes) and house sparrows (22 genes) (see the Table) had orthologs among the 270 genes identified based on GWA studies in humans and presented in Suppl. Material 3.

The study in C. elegans (Cook et al., 2016) produced 9 candidate genes (see the Table and Suppl. Material 11). One out of nine genes, pot-2, is orthologous to POT1 encoding a shelterin complex component in humans. The authors assume that another gene ZK1127.4 may also be involved in telomere length regulation, because BCCIP encoded by an orthologous human gene interacts with BRCA2 involved in DNA replication

In general, when comparing sets of candidate genes identified in humans and three other animal species, almost no orthologous genes are detected, which may be due to speciesspecific features of telomere length regulation, some peculiarities of regulation at various ontogenetic stages, and differences in sampled tissues or gender of the studied individuals.

## Conclusion

In the present paper, a compilation of telomere length-associated genes identified based on GWA studies and including the data on 270 human genes (see Suppl. Material 3), as well as 23, 22, and nine genes identified in cattle, house sparrow, and nematode (see the Table) is presented. The analysis of functions of 52 human genes with functional interpretation available (see Fig. 4, Suppl. Material 4) showed that telomere length may be affected by variants of genes encoding: (1) the structural components of telomerase; (2) the protein components of telomeric chromosome regions (shelterin complex and CST complex); (3) the proteins involved in telomerase biogenesis and regulating its activity; (4) the proteins regulating functional activity of shelterin subunits; (5) the proteins involved in telomere replication and/or capping; (6) the proteins controlling the alternative telomere lengthening pathway; (7) DNA damage response and repair proteins; (8) RNA exosome components

Candidate human genes identified by several research groups in population samples of different ethnic origin are determined: genes encoding telomerase components (TERC and TERT ) and STN1 encoding a subunit of CST complex (see Fig. 5). It seems that polymorphic loci that affect the functions of these genes can potentially be the most reliable predisposition markers for telomere length-associated diseases.

Comparison of the data obtained from GWA studies in humans (see Suppl. Material 3) with the results of similar experiments obtained for other animal species (see the Table and Suppl. Materials 9–11) confirmed and expanded the understanding of the complex polygenic nature of telomere length regulation. In addition, a pair of orthologous genes encoding a shelterin protein (POT1 in humans and pot-2 in C. elegans) was identified; this finding demonstrates the high biological significance of this gene in various species.

Systematized data on genes and their functions may lay the foundation for development of prognostic criteria for human pathologies explicitly associated with telomere length. In addition, the data on biological processes affecting telomere length and genes regulating these processes may be used for marker-assisted and genomic selection of the farm animals aimed at increasing the duration of their productive lifetime

## Conflict of interest

The authors declare no conflict of interest.
